# Maintaining healthy sleep patterns and frailty transitions: a prospective Chinese study

**DOI:** 10.1186/s12916-022-02557-0

**Published:** 2022-10-21

**Authors:** Yunqing Zhu, Junning Fan, Jun Lv, Yu Guo, Pei Pei, Ling Yang, Yiping Chen, Huaidong Du, Feifei Li, Xiaoming Yang, Daniel Avery, Junshi Chen, Zhengming Chen, Canqing Yu, Liming Li, on behalf of the China Kadoorie Biobank Collaborative Group, Robert Clarke, Rory Collins, Richard Peto, Robin Walters, Derrick Bennett, Ruth Boxall, Sue Burgess, Ka Hung Chan, Yumei Chang, Johnathan Clarke, Robert Clarke, Ahmed Edris Mohamed, Zammy Fairhurst-Hunter, Hannah Fry, Simon Gilbert, Alex Hacker, Mike Hill, Michael Holmes, Pek Kei Im, Andri Iona, Maria Kakkoura, Christiana Kartsonaki, Rene Kerosi, Kuang Lin, Mohsen Mazidi, Iona Millwood, Sam Morris, Qunhua Nie, Alfred Pozarickij, Paul Ryder, Saredo Said, Sam Sansome, Dan Schmidt, Paul Sherliker, Rajani Sohoni, Becky Stevens, Iain Turnbull, Robin Walters, Lin Wang, Neil Wright, Pang Yao, Yu Guo, Xiao Han, Can Hou, Jun Lv, Pei Pei, Chao Liu, Qingmei Xia, Zengchang Pang, Ruqin Gao, Shanpeng Li, Haiping Duan, Shaojie Wang, Yongmei Liu, Ranran Du, Yajing Zang, Liang Cheng, Xiaocao Tian, Hua Zhang, Yaoming Zhai, Feng Ning, Xiaohui Sun, Silu Lv, Junzheng Wang, Wei Hou, Wei Sun, Shichun Yan, Xiaoming Cui, Chi Wang, Zhenyuan Wu, Yanjie Li, Quan Kang, Huiming Luo, Tingting Ou, Xiangyang Zheng, Zhendong Guo, Shukuan Wu, Yilei Li, Huimei Li, Ming Wu, Yonglin Zhou, Jinyi Zhou, Ran Tao, Jie Yang, Jian Su, Fang Liu, Jun Zhang, Yihe Hu, Yan Lu, Liangcai Ma, Aiyu Tang, Shuo Zhang, Jianrong Jin, Jingchao Liu, Mei Lin, Zhenzhen Lu, Lifang Zhou, Changping Xie, Jian Lan, Tingping Zhu, Yun Liu, Liuping Wei, Liyuan Zhou, Ningyu Chen, Yulu Qin, Sisi Wang, Xianping Wu, Ningmei Zhang, Xiaofang Chen, Xiaoyu Chang, Mingqiang Yuan, Xia Wu, Xiaofang Chen, Wei Jiang, Jiaqiu Liu, Qiang Sun, Faqing Chen, Xiaolan Ren, Caixia Dong, Hui Zhang, Enke Mao, Xiaoping Wang, Tao Wang, Xi Zhang, Kai Kang, Shixian Feng, Huizi Tian, Lei Fan, Xiao Lin Li, Huarong Sun, Pan He, Xukui Zhang, Min Yu, Ruying Hu, Hao Wang, Xiaoyi Zhang, Yuan Cao, Kaixu Xie, Lingli Chen, Dun Shen, Xiaojun Li, Donghui Jin, Li Yin, Huilin Liu, Zhongxi Fu, Xin Xu, Hao Zhang, Jianwei Chen, Yuan Peng, Libo Zhang, Chan Qu

**Affiliations:** 1 Department of Epidemiology & Biostatistics, School of Public Health, Peking University, 100191 Beijing, China; 2 Peking University Center for Public Health and Epidemic Preparedness & Response, Beijing, 100191 China; 3 Fuwai Hospital, Chinese Academy of Medical Sciences, Beijing, 100037 China; 4 Chinese Academy of Medical Sciences, Beijing, 100730 China; 5 Medical Research Council Population Health Research Unit at the University of Oxford, Oxford, OX3 7LF UK; 6 Clinical Trial Service Unit & Epidemiological Studies Unit (CTSU), Nuffield Department of Population Health, University of Oxford, Oxford, OX3 7LF UK; 7 NCDs Prevention and Control Department, Qingdao CDC, Qingdao, 266033 Shandong China; 8 China National Center for Food Safety Risk Assessment, Beijing, 100022 China

**Keywords:** Sleep patterns, Frailty, Prospective cohort study

## Abstract

**Background:**

Little is known about the effects of maintaining healthy sleep patterns on frailty transitions.

**Methods:**

Based on 23,847 Chinese adults aged 30–79 in a prospective cohort study, we examined the associations between sleep patterns and frailty transitions. Healthy sleep patterns included sleep duration at 7 or 8 h/d, without insomnia disorder, and no snoring. Participants who persisted with a healthy sleep pattern in both surveys were defined as maintaining a healthy sleep pattern and scored one point. We used 27 phenotypes to construct a frailty index and defined three statuses: robust, prefrail, and frail. Frailty transitions were defined as the change of frailty status between the 2 surveys: improved, worsened, and remained. Log-binomial regression was used to calculate the prevalence ratio (PR) to assess the effect of sleep patterns on frailty transitions.

**Results:**

During a median follow-up of 8.0 years among 23,847 adults, 45.5% of robust participants, and 10.8% of prefrail participants worsened their frailty status, while 18.6% of prefrail participants improved. Among robust participants at baseline, individuals who maintained sleep duration of 7 or 8 h/ds, without insomnia disorder, and no-snoring were less likely to worsen their frailty status; the corresponding PRs (95% CIs) were 0.92 (0.89–0.96), 0.76 (0.74–0.77), and 0.85 (0.82–0.88), respectively. Similar results were observed among prefrail participants maintaining healthy sleep patterns. Maintaining healthy sleep duration and without snoring, also raised the probability of improving the frailty status; the corresponding PRs were 1.09 (1.00–1.18) and 1.42 (1.31–1.54), respectively. Besides, a dose-response relationship was observed between constantly healthy sleep scores and the risk of frailty transitions (*P* for trend < 0.001).

**Conclusions:**

Maintaining a comprehensive healthy sleep pattern was positively associated with a lower risk of worsening frailty status and a higher probability of improving frailty status among Chinese adults.

**Supplementary Information:**

The online version contains supplementary material available at 10.1186/s12916-022-02557-0.

## Background

Frailty, which usually occurs in the elderly, can increase vulnerability due to the decline in function and energy reserve of multi-organ systems. It also affects young adults with chronic diseases or other disorders [1, 2]. Frailty status can worsen due to biological aging or illness and improve due to body recovery. Besides, maintaining a healthy lifestyle could also improve the frailty status. A recent study based on China Kadoorie Biobank (CKB) reported that adherence to a healthy lifestyle (i.e., nonsmoking, nonheavy alcohol drinking) could lower the risk of frailty worsening [3].

Nowadays, people who complain about poor sleep quality have been getting younger. Unfortunately, young adults are more vulnerable to acute and chronic sleep deficiency than older adults [4]. Previous prospective studies explored the associations between sleep behaviors at baseline and the subsequent frailty status. However, the effects of unhealthy sleep duration were still inconclusive [5–7]. A prospective study with a small sample reported the association between sleep difficulties and worsening frailty [8]. Few studies focused on the effect of snoring, a more common sleep problem in adults, which was associated with risks of major chronic diseases [9, 10]. In addition, sleeping patterns, consisting of behaviors above, their overall effect on frailty transitions was unclear yet. Besides, evidence about the impact of maintaining healthy sleep patterns on frailty transitions was scarce, and no studies examined the associations among young and middle-aged adults.

Therefore, we conducted the present study based on the large population-based cohort, aimed to examine the effect of different sleep patterns and maintaining healthy sleep patterns on frailty transitions, and the dose-response relationship between healthy sleep scores and frailty transitions among middle-aged adults and the elderly.

## Methods

### Study design and participants

The CKB study is a prospective cohort study that recruited 512,725 adults aged 30–79 years living in 10 regions across China. Extensive questionnaire data, physical measurements, and blood samples were collected upon baseline assessment in 2004–2008, led by trained health workers. Besides, periodic resurveys were conducted in 2008 and 2013–2014 in a random sample of 5% surviving participants. The second resurvey added several detailed items in the questionnaire and physical examination. Details of CKB design have been described previously [11]. The CKB study was approved by the Ethics Review Committee of the Chinese Center for Disease Control and Prevention (Beijing, China, 005/2004) and the Oxford Tropical Research Ethics Committee, University of Oxford (Cambridge, United Kingdom, 025-04). All participants provided written informed consent in the CKB study.

In the present study, we included the participants who completed both the baseline and second resurvey without missing values on the variables for frailty index (FI) construction. We excluded frail participants at baseline from the present analyses to avoid reverse causality.

### Assessment of sleep pattern

Detailed information on sleep patterns and other covariates was collected through a laptop-based questionnaire at baseline and resurvey, including sociodemographic characteristics (age, sex, study areas, highest education), smoking status, and alcohol consumption.

Questionnaire for sleep patterns in CKB surveys was shown in the Additional file 1: Text S2. The present study defined the following sleep patterns. (a) *Short/long habitual sleep duration*: participants were asked, “How many hours do you typically sleep per day (including naps)?” Sleep duration could only be reported on an hourly basis. Short sleep duration was defined as 6 h/d or less, while long sleep duration was defined as 9 h or more, according to the American National Sleep Foundation [12]. (b) *Insomnia disorder*: participants were asked for the insomnia symptoms for at least 3 days/week in the past month, which were having difficulties in initiating or maintaining sleep (DIMS), early morning awakening (EMA), daytime dysfunction (DDF), and having to take medicine (herbal or sleeping pills) at least once a week to help sleep. According to the Diagnostic and Statistical Manual of Mental disorders (version 4) (DSM-4), those who reported either DIMS or EMA and reported DDF, or those who reported taking medicines to help sleep, were classified as having insomnia disorder [13, 14]. (c) *Snoring*: participants self-reported their usual snoring status during sleep as often, sometimes, and never/I do not know. Those who chose the first two options were classified into the snoring group, and others were in the no-snoring group. In our study, sleep duration (Spearman correlation coefficient = 0.63) and snoring (weighted-Kappa = 0.69) showed good reproducibility among 15,720 participants who completed a repeated questionnaire survey within 1~2 weeks after baseline. (d) *Baseline healthy sleep score*s: Previously, Fan and the colleagues developed an index score for healthy sleep pattern from multiple sleep behaviors (i.e., sleep duration, insomnia, snoring). It was replicated independently in a large cohort [15, 16]. In our study, participants were given one point for each of the three sleep patterns at baseline (sleep duration: 7 or 8 h/d, without insomnia disorder, without snoring). (e) *Maintaining healthy sleep patterns*: For each sleep pattern, participants were categorized into 2 groups (yes or no) according to whether the participants maintained the healthy sleep pattern both at the baseline and the second resurvey and were assigned 1 or 0 points for each sleep pattern. The total constantly healthy sleep scores were the number of maintaining healthy sleep patterns.

### FI construction and frailty transitions

Frailty index (FI) was one of the widely used measures of frailty status, which was developed by Mitnitski and colleagues [17]. FI included multiple health deficits across diverse physiological systems. Based on CKB baseline survey, we have constructed FI following a standard procedure, in which 28 deficits were selected. And the accelerated aging, measured by the FI, was associated with the risk of mortality [18]. The deficits included 14 self-reported diagnoses of diseases (i.e., coronary heart disease, diabetes, cancer), 10 self-reported symptoms (i.e., insomnia symptoms, frequently cough, bowel movements), and 4 physical measurements (i.e., body mass index [BMI], waist-to-hip-ratio [WHR], heart rate) [18]. Among them, BMI (kg/m^2^) was calculated by dividing weight (kg) by the square of standing height (m), WHR was the ratio of waist circumference to hip circumference, and daily physical activity was calculated by multiplying the metabolic equivalent of tasks (METs) for each kind of activity by the number of hours spent on the corresponding activity per day and then summing up the MET-hours for all activities [19].

The FI construction for the second resurvey was slightly different on disease history. For example, chronic heart disease (CHD) was included in the baseline survey, but in more detailed categories in the second resurvey, including acute myocardial infarction, angina, or other ischemic heart diseases, individuals with any of the 3 subtypes were categorized as the CHD patients. In addition, we included the incident cases during the follow-up period. All other variables were measured in the same way.

In the present study, we removed the insomnia symptoms from the construction of FI, leaving 27 items in the FI construction for both surveys. Then, we cut frailty into three statuses according to FI: robust (FI ≤ 0.10), prefrail (0.10 < FI < 0.25), and frail (≥ 0.25) [18]. Accordingly, the frailty transitions were defined by the change of frailly status from baseline to the second resurvey, which included robust remained, robust worsening (from robust to prefrail or frail), prefrail remained, prefrail improvement (from prefrail to robust), and prefrail worsening (from prefrail to frail).

### Statistical analysis

The present study first described the baseline characteristics and sleep patterns by frailty transitions, adjusting for sex, age, and study area. Then, we examined the associations between baseline sleep patterns and frailty transitions. Participants who remained in their frailty status at baseline were set as the reference group, respectively (i.e., robust remained vs. worsening, prefrail remained vs. improved, prefrail remained vs. worsening). Considering that the odds ratio may overestimate the effect with high-prevalence outcomes [20], we used log-binomial regression to estimate the prevalence ratios (PR) and 95%CI [21], adjusting for sex, age, study area (urban or rural), highest education (higher than middle school or not), smoking status (current smoker or not), and alcohol consumption (daily drinker or not).

Furthermore, we examined whether maintaining healthy sleep patterns was associated with frailty transitions. Besides, we assessed the effects of baseline and constantly healthy sleep scores on the frailty transitions and tested their linear trend by treating the scores as continuous variables. All models were log-binomial regression with the same adjustment of the covariates above. In addition, considering the differences in sleep patterns and frailty status between older and younger participants [12, 22], we performed the sensitivity analyses by examining the associations above among those aged < 60 (*n* = 18,995). Besides, we further adjusted for the major diseases and medication statuses at baseline (had disease and medication, had disease but without medication, without disease) separately for cardiovascular diseases (including coronary heart disease, stroke or transient ischemic attack, hypertension) and their medications (including aspirin, angiotensin-converting enzyme inhibitors, *β*-blocker, diuretics, statins, and calcium antagonists) and diabetes and its medications (including chlorpropamide, metformin, and insulin) in the sensitivity analysis. Statistical analysis was performed in Stata 16.0, and the graphs were plotted with R 4.0.5. Two-tailed *P* < 0.05 indicated statistical significance.

## Results

### Descriptive analysis

**Fig. 1:**
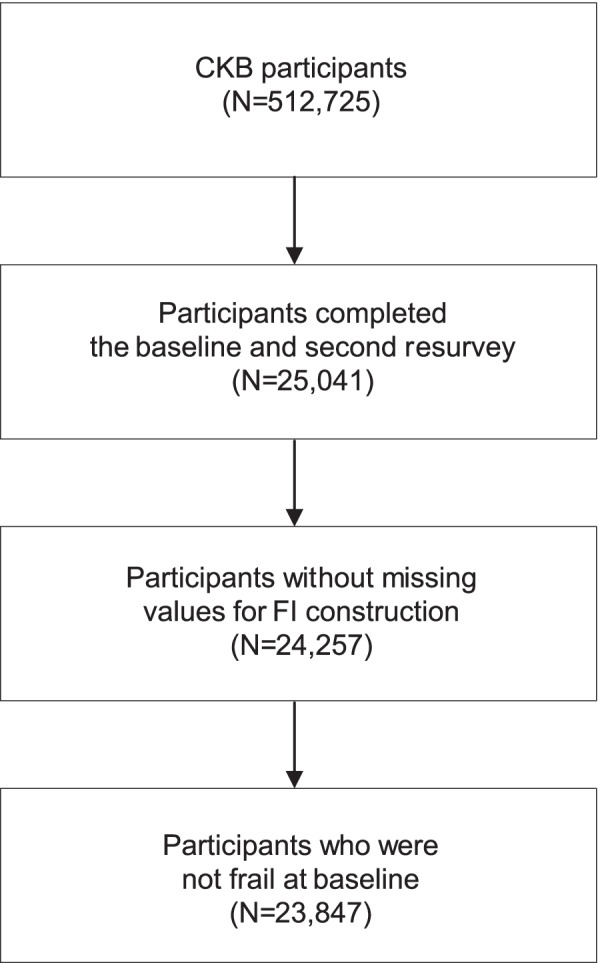
Sample selection of the study. CKB, China Kadoorie Biobank; FI, frailty index

Among the 512,725 CKB participants, 25,041 completed both the baseline and second resurvey. After excluding those with missing values for FI construction (1 missed BMI, 4 missed WHR, 779 missed forced vital capacity) or those who were frail at baseline (*n* = 410), a total of 23,847 were included in this study (Fig. 1).

During the median 8.0 (interquartile range: 7.4–8.6) years follow-up, 45.5% had worsening frailty status in the secondary resurvey among the 14,325 robust participants at baseline. Of the 9522 baseline prefrail participants, 10.8% became frail, and 18.6% became robust in the second resurvey.

Compared with participants who remained in their baseline frailty status, those who worsened were more likely to be elder, urban residents, more likely to have cardiovascular diseases or diabetes and the corresponding medication, and more likely to have long or short sleep duration at baseline. In contrast, participants who improved their frailty status were more likely to be younger, rural residents, more likely to sleep 7~8 h/d, and more unlikely to snore during sleep at baseline (*P* < 0.05) (Table 1).

**Table 1:** Baseline characteristics of 23,847 participants by transitions of frailty

Baseline characteristics	Baseline robust			Baseline prefrail			
	Remained	Worsening	***P***	Improvement	Remained	Worsening	***P***
***N*** **(%)**	7808 (54.5)	6517 (45.5)		1775 (18.6)	6716 (70.5)	1031 (10.8)	
**Sociodemographic**							
Male	38.9	38.3	0.485	40.6	37.8	33.0	< 0.001
Urban	41.3	44.3	< 0.001	38.5	43.9	54.7	< 0.001
Age (years)	46.7	51.4	< 0.001	50.4	55.1	59.1	< 0.001
Lower than middle school	46.8	49.5	< 0.001	59.0	58.1	59.0	0.975
**Lifestyle factors**							
Current smoker	26.4	28.4	0.001	26.8	27.3	29.5	0.068
Current drinker	52.7	53.1	0.028	50.5	49.5	46.9	0.597
**Diseases status and medication**							
Had cardiovascular diseases and medication	0.9	1.9	< 0.001	4.4	8.6	10.4	< 0.001
Had diabetes and medication	0.2	0.5	0.009	0.8	3.2	5.4	< 0.001
**Sleep duration (h/d)**			0.001				< 0.001
7 or 8	64.5	61.9		60.9	58.8	52.0	
≤ 6	18.7	20.6		21.7	23.5	30.4	
≥ 9	16.8	17.5		17.4	17.8	17.7	
**Insomnia**			0.168				0.001
No	98.3	97.9		95.6	95.6	92.6	
Yes	1.7	2.1		4.4	4.4	7.4	
**Snoring**			< 0.001				< 0.001
No	61.8	54.8		52.5	43.9	42.6	
Yes	38.2	45.2		47.5	56.1	57.4	
**Baseline healthy sleep scores**			< 0.001				< 0.001
0~1	14.0	17.7		20.0	24.1	30.7	
2	47.0	49.3		49.5	52.1	48.7	
3	39.0	33.0		30.4	23.8	20.6	

Baseline characteristics were adjusted for sex, age, and study areas, except for the 3 variables

### Associations between sleep patterns and frailty transitions

For the participants robust at baseline, short sleep duration had a 5% increased risk (95% CI: 1.01–1.10) of worsening frailty status in the secondary resurvey than those who slept 7 or 8 h/d at baseline; those who snored at baseline had 13% (95% CI: 1.09–1.17) increased risk of worsening frailty status than those who did not snore, while among the participants prefrail at baseline, those who slept 6 h/d or less or those with insomnia disorder at baseline had 40% (95% CI: 1.23–1.58) and 53% (95% CI: 1.23–1.89) increased risk of frailty in the secondary resurvey respectively, and those who snored at baseline were less likely to improve their frailty status (PR = 0.78, 95% CI: 0.71–0.84) (Table 2). Similar associations were observed among individuals aged < 60 and in analysis additionally adjusting for major diseases and medication statuses at baseline (see Additional file 1: Table S1, Table S2).

**Table 2:** Associations between baseline sleep patterns and frailty transitions

Baseline sleep patterns	Baseline robust		Baseline prefrail			
	Worsening (%)	PR (95%CI)	Worsening (%)	PR (95%CI)	Improvement (%)	PR (95%CI)
**Sleep duration (h/d)**						
7 or 8	44.0	1.00	9.5	1.00	19.5	1.00
≤ 6	51.5	1.05 (1.01–1.10)	15.2	1.40 (1.23–1.58)	15.8	0.95 (0.85–1.06)
≥ 9	44.0	1.03 (0.98–1.08)	9.5	1.07 (0.90–1.26)	19.6	0.94 (0.85–1.04)
**Insomnia**						
No	45.4	1.00	10.6	1.00	18.6	1.00
Yes	50.2	1.08 (0.97–1.20)	15.7	1.53 (1.23–1.89)	19.1	1.05 (0.87–1.26)
**Snoring**						
No	41.8	1.00	10.2	1.00	21.6	1.00
Yes	50.7	1.13 (1.09–1.17)	11.3	1.07 (0.95–1.20)	16.2	0.78 (0.71–0.84)

The multivariable model was adjusted for sex, age, study area, highest education, smoking status, and alcohol consumption

*Abbreviations*: *PR* prevalence ratio

As to constantly healthy status, constantly healthy sleep duration (7 or 8 h/d), constantly without insomnia disorder, and constantly no-snoring of baseline robust participants, were associated with 8% (PR = 0.92, 95% CI: 0.89–0.96), 24% (PR = 0.76, 95% CI: 0.74–0.77), and 15% (PR = 0.85, 95% CI: 0.82–0.88) lower risk of worsening frailty status. Among the baseline prefrail participants, constantly healthy sleep duration (PR = 0.74, 95% CI: 0.65–0.84) and without insomnia disorder (PR = 0.27, 95% CI: 0.25–0.29) could lower the risk of worsening frailty status. Besides, constantly healthy sleep duration and no snoring could increase the possibility of improving the frailty status of baseline prefrail participants (Table 3). Associations for sensitivity analysis adjusting for major diseases and medication statuses at baseline were similar to the results above (see Additional file 1: Table S3), while constantly healthy sleep duration was not associated with improving the frailty status among those younger than 60 years old (see Additional file 1: Table S4).

**Table 3:** Associations between constantly healthy sleep patterns and frailty transitions

Constantly healthy sleep patterns	Baseline robust		Baseline prefrail			
	Worsening (%)	PR (95%CI)	Worsening (%)	PR (95%CI)	Improvement (%)	PR (95%CI)
**Constantly healthy Sleep duration (7 or 8h/d)**						
No	48.2	1.00	12.2	1.00	17.5	1.00
Yes	41.5	0.92 (0.89–0.96)	8.3	0.74 (0.65–0.84)	20.7	1.09 (1.00–1.18)
**Constantly without insomnia disorder**						
No	58.8	1.00	19.0	1.00	16.1	1.00
Yes	43.8	0.76 (0.74–0.77)	9.4	0.27 (0.25–0.29)	19.1	1.12 (0.99–1.26)
**Constantly no**–**snoring**						
No	49.3	1.00	11.3	1.00	16.5	1.00
Yes	40.3	0.85 (0.82–0.88)	9.9	0.90 (0.79–1.02)	23.3	1.42 (1.31–1.54)

Constantly healthy sleep patterns mean maintaining the healthy sleep patterns at baseline and secondary resurvey. The multivariable model was adjusted for sex, age, study area, highest education, smoking status, and alcohol consumption

*Abbreviations*: *PR* prevalence ratio

### Dose-response relationship between sleep scores and frailty transitions

**Fig. 2:**
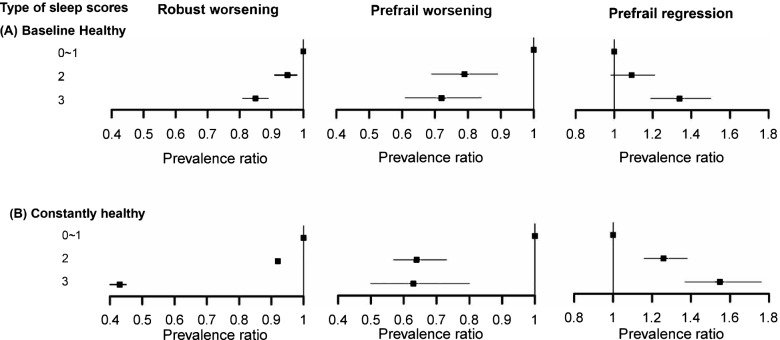
Dose-response Relationships between sleep scores and frailty transitions. The forest plots showed the relationships between baseline (panel A), constantly healthy sleep scores (panel B), and robust worsening (left panel), prefrail worsening (middle panel), prefrail regression (right panel), which were with the semi-log horizontal axis. Multivariable model was adjusted for sex, age, study area, highest education, smoking status, and alcohol consumption. *P* for trend were all < 0.001

We observed a dose-response relationship between sleep scores and the frailty transitions. With the increasing baseline healthy sleep scores, the risk of robust or prefrail worsening decreased, and the possibility of prefrail improvement increased (*P* linear trend < 0.001). Among participants robust at baseline, compared with those who scored 0~1 point, the PRs (95%CI) of worsening the frailty status were 0.95 (0.91–0.98) and 0.85 (0.81–0.89) for those who scored 2, 3 points, respectively. Similar results were observed in prefrail worsening participants. While among participants prefrail at baseline, compared with those who scored 0~1 points, the corresponding PRs (95%CI) of improving the frailty status were 1.09 (0.98–1.21) and 1.34 (1.19–1.50) for those who scored 2 and 3 points (Fig. 2A, see Additional file 1: Table S5).

Consistent with baseline healthy sleep scores, dose-response relationships were observed between constantly healthy sleep scores and the risk of frailty transitions during follow-up (all *P* linear trend < 0.001) (Fig. 2B, see Additional file 1: Table S5). Furthermore, the beneficial effect of constantly healthy sleep scores was larger than that of the baseline scores. Besides, we observed similar dose-response relationships for those aged < 60 years and in the analysis adjusting for major diseases and medication statuses at baseline (see Additional file 1: Table S1, S2, S3 and S4).

## Discussion

In the present study, we found that sleep behaviors, including sleep duration ≤ 6 h/d, insomnia, or snoring, increased the risk of worsening the frailty status, while snoring decreased the possibility of improving the prefrail status. Maintaining healthy sleep patterns could prevent the worsening of frailty status and improve their frailty status. Such associations showed in dose-response relationships for both baseline and constantly healthy sleep scores.

Existing cross-sectional studies showed mixed results on the relationship between sleep duration and frailty [23–25]. Few studies explored relationships in prospective cohort study design. A Mexican cohort study of 309 older adults reported that participants who slept less than 5 h/d had a higher risk of frailty at 4.4 years of follow-up (RR = 1.80, 95% CI: 1.04–3.11) [5]. A Spanish prospective study of 1309 older adults demonstrated that adequate sleep duration (7~8 h/d) was associated with a substantial reduction in the risk of frailty [7]. Our study observed similar results. Short sleep duration could be an early marker of poor health status (such as heart disease or obesity). Such poor condition leads to an increased risk of frailty [26]. Though we did not observe the association between long sleep duration and frailty, some previous studies reported an adverse effect of long sleep duration; the possible reason was that they focused on cognitive frailty, which was different from physical frailty in the present study [5, 6, 27].

The associations between insomnia and frailty were consistent in both prospective and cross-sectional studies [25, 28, 29]. A prospective study of 14,208 older adults used sleep medication as a proxy for insomnia disorder and reported an increased risk of frailty (hazard ratio [HR] = 1.35,95% CI:1.13–1.61) [30]. Besides, another prospective study of 3844 older adults showed a bidirectional relationship between insomnia and frailty, which indicated that insomnia might be an early index of frailty [28].

Snoring was reported as an unhealthy sleep behavior among Chinese and western population. Participants with habitual snoring had increased risks of cardiovascular diseases [9, 31] and diabetes [32], due to the hypoxia caused by stenosis of the upper airway [33], the process of atherosclerosis led by large swings in pleural pressure during snoring [34], while only one prospective cohort study (3220 Korean women) focused on the associations between snoring and frailty. Consistent with us, it found that snoring increased the risk of frailty (HR = 1.68, 95% CI: 1.16–2.43) [35].

The present study was the largest prospective study on the relationship between transitions of sleep patterns and frailty status. Previous studies with small sample sizes have considered frailty transitions during follow-up. A prospective study based on National Social Life, Health and Aging Project, with a 5-year follow-up (*n* = 615), adjusted for the baseline frailty status when examining the associations between sleep fragmentation and the frailty [36]. While another cohort study from 2013 to 2018 (*n* = 306) calculated the frailty transition between the two waves of their study, they only found out that sleep difficulties were positively associated with the transition from robust to prefrail status (odds ratio [OR] = 1.76, 95% CI: 1.07–2.90) [8]. In addition, the present study used healthy sleep scores to integrate multiple dimensions of sleep behaviors and observed the cumulative effects on frailty transitions. Such beneficial effects of constantly healthy sleep scores were more prominent than baseline, highlighting the importance of maintaining healthy sleep patterns for frailty prevention.

The biological mechanisms by which sleep affects frailty were complex. Short sleep duration or insomnia caused an immediate reduction of testosterone levels and therefore resulted in oxidative stress and chronic inflammation. Also, it might aggravate the hormone secretion imbalance, which is suggested to be related to frailty [5, 37, 38]. While snoring caused the interruptions of breathing during sleep, leading to the disruption of rapid-eye-movement sleep and nocturnal hypoxemia, the latter was suggested to be a pathway of sympathetic activation and frailty [39, 40].

To our best knowledge, the present study was the first to examine the association between maintaining healthy sleep patterns and the frailty transitions between the two waves of a large cohort study in Chinese adults. Our results showed the benefit of maintaining healthy sleep patterns for participants with different frailty statuses, for it could not only lower the risk of worsening frailty status, but it could also elevate the probability of improving frailty status. In addition, our study assessed comprehensively sleep behaviors in a broader age span, as sleep problems were common among young adults. Besides, the dose-response relationship in both baseline and maintaining healthy sleep with frailty transitions highlighted the cumulative effect of different sleep patterns on frailty transition. However, our study had several limitations. First, the CKB study did not evaluate the cognitive function deficit at baseline,; therefore, we could not construct the FI representing cognitive decline. Second, although questions for sleep patterns were from standardized questionnaires [13, 14], these questions had not been validated in our population, which might lead to the possibility of misclassification. However, measurement errors may be non-differential in prospective study design, and the associations were more likely to be biased toward the null, while further research using validated activity monitors and other devices was necessary to measure sleep behaviors objectively. Third, considering the fluctuation of the insomnia symptoms, we only collected the symptoms within one month might not be long enough to capture participants with chronic insomnia disorder [41, 42]. Fourth, we did not measure some variables relevant to potential biological mechanisms and potential modification effects, such as hormone levels, and oxyhemoglobin saturation, we could not estimate their effects on the association between sleep and frailty. In addition, due to the nature of observational study, we could not estimate the causal relationship. Future Mendelian randomization studies were necessary for the causal inference. Last, only the survival participants could complete the secondary resurvey. The possible selection bias should be considered when we extend the findings to a broader population.

## Conclusions

In this prospective cohort study of Chinese adults, keeping healthy sleep patterns was positively associated with lower risks of frail and prefrail worsening and a higher probability of prefrail status improving. Such relationships were in a dose-response manner, especially for those maintaining healthy sleep patterns. Our study highlighted the importance of adherence to a long-term healthy sleep pattern to better frailty status.

## Supplementary Information

**Additional file 1: Text S1.** Members of the China Kadoorie Biobank Collaborative Group. **Text S2.** Questionnaire for the sleep patterns in the survey of China Kadoorie Biobank. **Table S1.** Associations between Baseline Sleep Patterns and Frailty Transitions among Participants Aged < 60. **Table S2.** Associations between Baseline Sleep Patterns and Frailty Transitions Adjusted for Major Diseases and Medication. **Table S3.** Associations between Constantly Healthy Sleep Patterns and Frailty Transitions Adjusted for Major Diseases and Medication. **Table S4.** Associations between Constantly Healthy Sleep Patterns and Frailty Transitions among Participants Aged < 60. **Table S5.** Associations between Sleep Scores and Frailty Transitions.

## Data Availability

The dataset supporting the conclusions of this article is available on study website (http://www.ckbiobank.org) for access policy and procedures.

## Abbreviations

CKB: China Kadoorie Biobank
FI: Frailty index
BMI: Body mass index
WHR: Waist-to-hip-ratio
FVC: Forced vital capacity
MET: Metabolic equivalent of tasks
PR: Prevalence ratio
