# Supplementary material for: Maintaining healthy sleep patterns and frailty transitions: a prospective Chinese study
Source: BMC Med. 2022 Oct 21;20:354. doi: 10.1186/s12916-022-02557-0 (PMC9585775; doi:10.1186/s12916-022-02557-0)
Supplement: Supplementary file 1 — Additional file 1: Text S1. Members of the China Kadoorie Biobank Collaborative Group. Text S2. Questionnaire for the sleep patterns in the survey of China Kadoorie Biobank. Table S1. Associations between Baseline Sleep Patterns and Frailty Transitions among Participants Aged < 60. Table S2. Associations between Baseline Sleep Patterns and Frailty Transitions Adjusted for Major Diseases and Medication. Table S3. Associations between Constantly Healthy Sleep Patterns and Frailty Transitions Adjusted for Major Diseases and Medication. Table S4. Associations between Constantly Healthy Sleep Patterns and Frailty Transitions among Participants Aged < 60. Table S5. Associations between Sleep Scores and Frailty Transitions. [file 12916_2022_2557_MOESM1_ESM.docx]

**Supplementary material**

**Table of Contents**

[**Additional file 1.** 2](#_Toc113357721)

[**Text S1.** Members of the China Kadoorie Biobank Collaborative Group. 2](#_Toc113357722)

[**Text S2.** Questionnaire for the sleep patterns in the survey of China Kadoorie Biobank. 4](#_Toc113357723)

[**Table S1.** Associations between Baseline Sleep Patterns and Frailty Transitions among Participants Aged < 60. 5](#_Toc113357724)

[**Table S2.** Associations between Baseline Sleep Patterns and Frailty Transitions Adjusted for Major Diseases and Medication. 6](#_Toc113357725)

[**Table S3.** Associations between Constantly Healthy Sleep Patterns and Frailty Transitions Adjusted for Major Diseases and Medication. 7](#_Toc113357726)

[**Table S4.** Associations between Constantly Healthy Sleep Patterns and Frailty Transitions among Participants Aged < 60 8](#_Toc113357727)

[**Table S5.** Associations between Sleep Scores and Frailty Transitions. 9](#_Toc113357728)

**Additional file 1.**

**Text S1.** Members of the China Kadoorie Biobank Collaborative Group.

**International Steering Committee:** Junshi Chen, Zhengming Chen (PI), Robert Clarke, Rory Collins, Yu Guo, Liming Li (PI), Jun Lv, Richard Peto, Robin Walters. **International Co-ordinating Centre, Oxford:** Daniel Avery, Derrick Bennett, Ruth Boxall, Sue Burgess, Ka Hung Chan, Yumei Chang, Yiping Chen, Zhengming Chen, Johnathan Clarke; Robert Clarke, Huaidong Du, Ahmed Edris Mohamed, Zammy Fairhurst-Hunter, Hannah Fry, Simon Gilbert, Alex Hacker, Mike Hill, Michael Holmes, Pek Kei Im, Andri Iona, Maria Kakkoura, Christiana Kartsonaki, Rene Kerosi, Kuang Lin, Mohsen Mazidi, Iona Millwood, Sam Morris, Qunhua Nie, Alfred Pozarickij, Paul Ryder, Saredo Said, Sam Sansome, Dan Schmidt, Paul Sherliker, Rajani Sohoni, Becky Stevens, Iain Turnbull, Robin Walters, Lin Wang, Neil Wright, Ling Yang, Xiaoming Yang, Pang Yao.

**National Co-ordinating Centre, Beijing:** Yu Guo, Xiao Han, Can Hou, Jun Lv, Pei Pei, Chao Liu, Canqing Yu, Qingmei Xia. **10 Regional Co-ordinating Centres: Qingdao CDC:** Zengchang Pang, Ruqin Gao, Shanpeng Li, Haiping Duan, Shaojie Wang, Yongmei Liu, Ranran Du, Yajing Zang, Liang Cheng, Xiaocao Tian, Hua Zhang, Yaoming Zhai, Feng Ning, Xiaohui Sun, Feifei Li. **Licang CDC:** Silu Lv, Junzheng Wang, Wei Hou. **Heilongjiang Provincial CDC:** Wei Sun, Shichun Yan, Xiaoming Cui. **Nangang CDC:** Chi Wang, Zhenyuan Wu,Yanjie Li, Quan Kang. **Hainan Provincial CDC:** Huiming Luo, Tingting Ou. **Meilan CDC:** Xiangyang Zheng, Zhendong Guo, Shukuan Wu, Yilei Li, Huimei Li. **Jiangsu Provincial CDC:** Ming Wu, Yonglin Zhou, Jinyi Zhou, Ran Tao, Jie Yang, Jian Su. **Suzhou CDC:** Fang Liu, Jun Zhang, Yihe Hu, Yan Lu, Liangcai Ma, Aiyu Tang, Shuo Zhang, Jianrong Jin, Jingchao Liu. **Guangxi Provincial CDC:** Mei Lin, Zhenzhen Lu. **Liuzhou CDC:** Lifang Zhou, Changping Xie, Jian Lan,Tingping Zhu,Yun Liu, Liuping Wei, Liyuan Zhou, Ningyu Chen, Yulu Qin, Sisi Wang. **Sichuan Provincial CDC:** Xianping Wu, Ningmei Zhang, Xiaofang Chen, Xiaoyu Chang. **Pengzhou CDC:** Mingqiang Yuan, Xia Wu, Xiaofang Chen, Wei Jiang, Jiaqiu Liu, Qiang Sun. **Gansu Provincial CDC:** Faqing Chen, Xiaolan Ren, Caixia Dong. **Maiji CDC:** Hui Zhang, Enke Mao, Xiaoping Wang, Tao Wang, Xi zhang. **Henan Provincial CDC:** Kai Kang, Shixian Feng, Huizi Tian, Lei Fan. **Huixian CDC:** XiaoLin Li, Huarong Sun, Pan He, Xukui Zhang. **Zhejiang Provincial CDC:** Min Yu, Ruying Hu, Hao Wang. **Tongxiang CDC**: Xiaoyi Zhang, Yuan Cao, Kaixu Xie, Lingli Chen, Dun Shen. **Hunan Provincial CDC:** Xiaojun Li, Donghui Jin, Li Yin, Huilin Liu, Zhongxi Fu. **Liuyang CDC:** Xin Xu, Hao Zhang, Jianwei Chen,Yuan Peng, Libo Zhang, Chan Qu.

**Text S2.** Questionnaire for the sleep patterns in the survey of China Kadoorie Biobank.

1. How many hours do you typically sleep per day (including naps)? ________ hours.
2. During the past month, did you have any of the following for ≥3 days each week?
   - Taking > 30 minutes to fall asleep after going to bed or waking up in the middle of the night. (Yes/No)
   - Waking up early and not being able to go back to sleep. (Yes/No)
   - Needing to take medicine (including herbal or sleeping pills) at least once a week to help sleep. (Yes/No)
   - Having difficulty staying alert while at work, eating or meeting people during daytime. (Yes/No)
3. Do you usually take a daytime nap? (Yes, usually / Yes, but only in summer / No)
4. Do you snore during sleep? (Yes, usually / Yes, sometimes / No or don’t know)

**Table S1.** Associations between Baseline Sleep Patterns and Frailty Transitions among Participants Aged < 60.

|  | **Robust worsening** | |  | **Prefrail worsening** | |  | **Prefrail regress** | |
| --- | --- | --- | --- | --- | --- | --- | --- | --- |
|  | **Worsening(%)** | **PR (95%CI)** |  | **Worsening(%)** | **PR (95%CI)** |  | **Improvement(%)** | **PR (95%CI)** |
| **Sleep duration(h/d)** |  |  |  |  |  |  |  |  |
| 7 or 8 | 40.9 | 1.00 |  | 6.9 | 1.00 |  | 23.2 | 1.00 |
| ≤6 | 47.3 | 1.07 (1.02-1.13) |  | 11.8 | 1.53 (1.28-1.83) |  | 20.2 | 0.96 (0.85-1.07) |
| ≥9 | 41.2 | 1.05 (0.99-1.11) |  | 7.0 | 1.14 (0.90-1.44) |  | 23.8 | 0.97 (0.87-1.09) |
| **Insomnia** |  |  |  |  |  |  |  |  |
| No | 42.1 | 1.00 |  | 7.8 | 1.00 |  | 22.6 | 1.00 |
| Yes | 44.9 | 1.05 (0.91-1.21) |  | 11.1 | 1.44 (1.05-1.96) |  | 23.8 | 1.07 (0.88-1.30) |
| **Snoring** |  |  |  |  |  |  |  |  |
| No | 38.8 | 1.00 |  | 7.8 | 1.00 |  | 26.2 | 1.00 |
| Yes | 47.1 | 1.08 (1.08-1.08) |  | 8.2 | 0.93 (0.79-1.10) |  | 19.7 | 0.79 (0.72-0.86) |
| **Baseline healthy sleep scores** |  |  |  |  |  |  |  |  |
| 0~1 | 48.8 | 1.00 |  | 10.2 | 1.00 |  | 20.0 | 1.00 |
| 2 | 43.8 | 0.64 (0.62-0.66) |  | 7.6 | 0.76 (0.63-0.91) |  | 21.4 | 1.04 (0.93-1.17) |
| 3 | 37.3 | 0.55 (0.53-0.57) |  | 6.8 | 0.75 (0.60-0.95) |  | 27.7 | 1.29 (1.13-1.46) |
| P for trend |  | <0.001 |  |  | 0.005 |  |  | <0.001 |

Abbreviations: PR prevalence ratio.

The multivariable model was adjusted for sex, age, study area, highest education, smoking status, and alcohol consumption.

**Table S2.** Associations between Baseline Sleep Patterns and Frailty Transitions Adjusted for Major Diseases and Medication.

|  | **Robust worsening** | |  | **Prefrail worsening** | |  | **Prefrail regress** | |
| --- | --- | --- | --- | --- | --- | --- | --- | --- |
|  | **Worsening(%)** | **PR (95%CI)** |  | **Worsening(%)** | **PR (95%CI)** |  | **Improvement(%)** | **PR (95%CI)** |
| **Sleep duration(h/d)** |  |  |  |  |  |  |  |  |
| 7 or 8 | 44.0 | 1.00 |  | 9.5 | 1.00 |  | 19.5 | 1.00 |
| ≤6 | 51.5 | 1.03 (1.03-1.03) |  | 15.2 | 1.42 (1.25-1.61) |  | 15.8 | 0.92 (0.83-1.02) |
| ≥9 | 44.0 | 1.02 (1.02-1.02) |  | 9.5 | 1.07 (0.91-1.26) |  | 19.6 | 0.94 (0.85-1.05) |
| **Insomnia** |  |  |  |  |  |  |  |  |
| No | 45.4 | 1.00 |  | 10.6 | 1.00 |  | 18.6 | 1.00 |
| Yes | 50.2 | 1.03 (1.03-1.03) |  | 15.7 | 1.54 (1.25-1.91) |  | 19.1 | 1.03 (0.86-1.25) |
| **Snoring** |  |  |  |  |  |  |  |  |
| No | 41.8 | 1.00 |  | 10.2 | 1.00 |  | 21.6 | 1.00 |
| Yes | 50.7 | 1.07 (1.07-1.07) |  | 11.3 | 1.04 (0.93-1.17) |  | 16.2 | 0.80 (0.73-0.88) |
| **Baseline healthy sleep scores** |  |  |  |  |  |  |  |  |
| 0~1 | 53.0 | 1.00 |  | 13.9 | 1.00 |  | 15.6 | 1.00 |
| 2 | 47.0 | 0.97 (0.97-0.97) |  | 10.3 | 0.78 (0.69-0.89) |  | 18.0 | 1.09 (0.99-1.22) |
| 3 | 40.2 | 0.91 (0.91-0.91) |  | 9.1 | 0.72 (0.62-0.85) |  | 22.9 | 1.32 (1.18-1.49) |
| P for trend |  | <0.001 |  |  | <0.001 |  |  | <0.001 |

Abbreviations: PR prevalence ratio.

The multivariable model was adjusted for sex, age, study area, highest education, smoking status, alcohol consumption, statuses of diseases and medication separately for cardiovascular diseases and diabetes at baseline.

**Table S3.** Associations between Constantly Healthy Sleep Patterns and Frailty Transitions Adjusted for Major Diseases and Medication.

|  | **Robust worsening** | |  | **Prefrail worsening** | |  | **Prefrail regress** | |
| --- | --- | --- | --- | --- | --- | --- | --- | --- |
|  | **Worsening**  **(%)** | **PR (95%CI)** |  | **Worsening**  **(%)** | **PR (95%CI)** |  | **Improvement**  **(%)** | **PR (95%CI)** |
| **Constantly healthy Sleep duration (7 or 8h/d)** |  |  |  |  |  |  |  |  |
| No | 48.2 | 1.00 |  | 12.2 | 1.00 |  | 17.5 | 1.00 |
| Yes | 41.5 | 0.96 (0.96-0.96) |  | 8.3 | 0.74 (0.65-0.84) |  | 20.7 | 1.10 (1.01-1.20) |
| **Constantly without insomnia disorder** |  |  |  |  |  |  |  |  |
| No | 58.8 | 1.00 |  | 19.0 | 1.00 |  | 16.1 | 1.00 |
| Yes | 43.8 | 0.87 (0.87-0.87) |  | 9.4 | 0.23 (0.21-0.24) |  | 19.1 | 1.12 (0.99-1.26) |
| **Constantly no-snoring** |  |  |  |  |  |  |  |  |
| No | 49.3 | 1.00 |  | 11.3 | 1.00 |  | 16.5 | 1.00 |
| Yes | 40.3 | 0.91 (0.91-0.91) |  | 9.9 | 0.92 (0.81-1.04) |  | 23.3 | 1.39 (1.28-1.51) |
| **Constantly healthy sleep scores** |  |  |  |  |  |  |  |  |
| 0~1 | 53.0 | 1.00 |  | 13.6 | 1.00 |  | 15.5 | 1.00 |
| 2 | 42.6 | 0.92 (0.92-0.92) |  | 8.1 | 0.33 (0.29-0.36) |  | 20.8 | 1.26 (1.15-1.37) |
| 3 | 35.0 | 0.84 (0.84-0.84) |  | 7.8 | 0.31 (0.25-0.39) |  | 26.2 | 1.54 (1.36-1.74) |
| P for trend |  | <0.001 |  |  | <0.001 |  |  | <0.001 |

Abbreviations: PR prevalence ratio.

The multivariable model was adjusted for sex, age, study area, highest education, smoking status, alcohol consumption, statuses of diseases and medication separately for cardiovascular diseases and diabetes at baseline.

**Table S4.** Associations between Constantly Healthy Sleep Patterns and Frailty Transitions among Participants Aged < 60

|  | **Robust worsening** | |  | **Prefrail worsening** | |  | **Prefrail regress** | |
| --- | --- | --- | --- | --- | --- | --- | --- | --- |
|  | **Worsening**  **(%)** | **PR (95%CI)** |  | **Worsening**  **(%)** | **PR (95%CI)** |  | **Improvement**  **(%)** | **PR (95%CI)** |
| **Constantly healthy Sleep duration (7 or 8h/d)** |  |  |  |  |  |  |  |  |
| No | 44.6 | 1.00 |  | 9.3 | 1.00 |  | 21.8 | 1.00 |
| Yes | 38.7 | 0.91 (0.87-0.94) |  | 5.7 | 0.64 (0.53-0.77) |  | 24.1 | 1.07 (0.97-1.16) |
| **Constantly without insomnia disorder** |  |  |  |  |  |  |  |  |
| No | 56.1 | 1.00 |  | 14.2 | 1.00 |  | 19.8 | 1.00 |
| Yes | 40.3 | 0.48 (0.47-0.49) |  | 6.8 | 0.49 (0.41-0.58) |  | 23.2 | 1.11 (0.98-1.26) |
| **Constantly no-snoring** |  |  |  |  |  |  |  |  |
| No | 46.0 | 1.00 |  | 8.4 | 1.00 |  | 19.7 | 1.00 |
| Yes | 36.9 | 0.60 (0.58-0.62) |  | 7.0 | 0.95 (0.79-1.14) |  | 29.4 | 1.44 (1.32-1.58) |
| **Constantly healthy sleep scores** |  |  |  |  |  |  |  |  |
| 0~1 | 49.6 | 1.00 |  | 10.6 | 1.00 |  | 19.2 | 1.00 |
| 2 | 39.2 | 0.60 (0.58-0.62) |  | 5.5 | 0.55 (0.46-0.66) |  | 24.6 | 1.23 (1.11-1.34) |
| 3 | 32.2 | 0.49 (0.46-0.53) |  | 4.8 | 0.55 (0.38-0.79) |  | 33.2 | 1.56 (1.37-1.78) |
| P for trend |  | <0.001 |  |  | <0.001 |  |  | <0.001 |

Abbreviations: PR prevalence ratio.

The multivariable model was adjusted for sex, age, study area, highest education, smoking status, and alcohol consumption.

**Table S5.** Associations between Sleep Scores and Frailty Transitions.

|  | **Baseline robust** | |  | **Baseline prefrail** | | | | |
| --- | --- | --- | --- | --- | --- | --- | --- | --- |
|  | **Worsening**  **(%)** | **PR (95%CI)** |  | **Worsening**  **(%)** | **PR (95%CI)** |  | **Improvement (%)** | **PR (95%CI)** |
| **Baseline healthy sleep score** |  |  |  |  |  |  |  |  |
| 0~1 | 53.0 | 1.00 |  | 13.9 | 1.00 |  | 15.6 | 1.00 |
| 2 | 47.0 | 0.95 (0.91-0.98) |  | 10.3 | 0.79 (0.69-0.89) |  | 18.0 | 1.09 (0.98-1.21) |
| 3 | 40.2 | 0.85 (0.81-0.89) |  | 9.1 | 0.72 (0.61-0.84) |  | 22.9 | 1.34 (1.19-1.50) |
| P for trend |  | <0.001 |  |  | <0.001 |  |  | <0.001 |
| **Constantly healthy  sleep score** |  |  |  |  |  |  |  |  |
| 0~1 | 53.0 | 1.00 |  | 13.6 | 1.00 |  | 15.5 | 1.00 |
| 2 | 42.6 | 0.92 (0.92-0.92) |  | 8.1 | 0.64 (0.57-0.73) |  | 20.8 | 1.26 (1.16-1.38) |
| 3 | 35.0 | 0.43 (0.40-0.45) |  | 7.8 | 0.63 (0.50-0.80) |  | 26.2 | 1.55 (1.37-1.76) |
| P for trend |  | <0.001 |  |  | <0.001 |  |  | <0.001 |

Abbreviations: PR prevalence ratio.

The multivariable model was adjusted for sex, age, study area, highest education, smoking status, and alcohol consumption.
